# Screening, Linkage to Care and Treatment of Hepatitis C Infection in Primary Care Setting in the South of Italy

**DOI:** 10.3390/life10120359

**Published:** 2020-12-18

**Authors:** Anna Citarella, Simona Cammarota, Francesca F. Bernardi, Carmine Coppola, Maria D’Antò, Marianna Fogliasecca, Elio Giusto, Mario Masarone, Angelo Salomone Megna, Carmine Sellitto, Rosa Servodio, Massimo Smaldone, Laura Staiano, Ugo Trama, Valeria Conti, Marcello Persico

**Affiliations:** 1 LinkHealth Health Economics, Outcomes & Epidemiology s.r.l., 80143 Naples, Italy; simona.cammarota@linkhealth.it (S.C.); marianna.fogliasecca@linkhealth.it (M.F.); 2 General Direction of Health Care & Regional Health System Coordination, Drug & Device Politics, Campania Region, 80143 Naples, Italy; bernardi.francesca.futura@gmail.com (F.F.B.); ugo.trama@regione.campania.it (U.T.); 3 Hepatology Unit, Department of Internal Medicine, Gragnano Hospital, 80054 Naples, Italy; c.coppola@aslnapoli3sud.it (C.C.); laura.staiano@libero.it (L.S.); 4 Hepatology Unit, Department of Internal Medicine, Santa Maria delle Grazie Hospital, 80078 Naples, Italy; maria.danto@aslnapoli2nord.it; 5 “Cooperativa Moscati” Medical Association, 07017 Salerno, Italy; giustoelio@tin.it; 6 Internal Medicine and Hepatology Unit, Department of Medicine and Surgery, University of Salerno, 84084 Salerno, Italy; mmasarone@unisa.it (M.M.); mpersico@unisa.it (M.P.); 7 AORN San Pio, Rummo Hospital, 82100 Benevento, Italy; angelo.salomonemegna@ao-rummo.it; 8 Department of Medicine, Surgery and Dentistry “Scuola Medica Salernitana”, University of Salerno, 84084 Salerno, Italy; csellitto@unisa.it (C.S.); vconti@unisa.it (V.C.); 9 “Samnium Medica” Soc. Cooperative, 82100 Benevento, Italy; roservod@tin.it; 10 “Progetto Leonardo” Medical Association, 80078 Naples, Italy; massimo.smaldone@gmail.com

**Keywords:** direct acting antivirals, eradication, barriers, primary care, proactive case-finding

## Abstract

Hepatitis C virus (HCV) infection remains a pressing public health issue. Our aim is to assess the linkage to care of patients with HCV diagnosis and to support the proactive case-finding of new HCV-infected patients in an Italian primary care setting. This was a retrospective cohort study of 44 general practitioners (GPs) who managed 63,955 inhabitants in the Campania region. Adults with already known HCV diagnosis or those with HCV high-risk profile at June 2019 were identified and reviewed by GPs to identify newly diagnosed of HCV and to assess the linkage to care and treatment for the HCV patients. Overall, 698 HCV patients were identified, 596 with already known HCV diagnosis and 102 identified by testing the high-risk group (2614 subjects). The 38.8% were already treated with direct-acting antivirals, 18.9% were referred to the specialist center and 42.3% were not sent to specialist care for treatment. Similar proportions were found for patients with an already known HCV diagnosis and those newly diagnosed. Given that the HCV infection is often silent, case-finding needs to be proactive and based on risk information. Our findings suggested that there needs to be greater outreach, awareness and education among GPs in order to enhance HCV testing, linkage to care and treatment.

## 1. Introduction

Hepatitis C virus (HCV) infection is a global health and economic problem due to its substantial impact on morbidity and mortality [1,2]. Overall, 30% of HCV patients chronically infected may progress to cirrhosis in their lifetime, whereas 3–8% of cirrhotic patients may develop hepatocellular carcinoma (HCC) [3,4]. The progression to cirrhosis is often clinically silent and, as a result, many HCV patients do not come forward until cirrhosis, liver decompensation or HCC occurs [5,6,7].

The advent of direct-acting antivirals (DAAs) has completely revolutionized the treatment of HCV infection, yielding sustained virologic response (SVR) rates, even in difficult-to-treat patients, such as patients with advanced liver disease or chronic kidney disease [8]. The achievement of SVR has the potential benefits of risk reduction of liver disease progression, leading to better long-term clinical outcomes and improved health-related quality of life [9]. On this basis, in 2016, the World Health Organization (WHO) introduced the global targets necessary for obtaining “HCV elimination” in developed countries by 2030, including a 90% reduction in new cases of chronic hepatitis C, a 65% reduction in hepatitis C-related deaths and the treatment of 80% of eligible individuals with chronic HCV infection [10]. To achieve these goals, considerable efforts are required to enhance HCV awareness and diagnosis rates in all countries with development of strategies to link diagnosed patients to treatment and care.

In Italy, after an initial phase characterized by the prioritization of the patients with more severe liver disease (i.e., cirrhosis), necessary to contain DAA treatment costs in relation to a large number of people to be treated, there is now a phase of universalization of the access to therapy to all infected patients, totally free of charge [11]. To fully benefit from this universalization and achieve WHO targets, special programs of micro-elimination have already started to identify the HCV infection in people who inject drugs (PWID), HIV-infected subjects, prisoners and the homeless [12,13,14]. On the other hand, with regards to the general population, there is still the need to optimize screening policies, increase the linkage to care and treatment uptake. In this light, general practitioners (GPs), as the first gatekeepers of the Italian public health system, could play a crucial role in the screening of individuals at risk through HCV testing and referring HCV positive patients to appropriate specialist care for further investigation and DAA treatment [15,16]. However, previous projects involving GPs and conducted in other countries suggest low GPs awareness of risk groups for HCV infection other than PWID, incomplete knowledge regarding treatment and whom to test and poor linkage to care [17,18]. 

The purpose of this study is to assess the linkage to care of patients with HCV diagnosis and to support the proactive case-finding of new HCV-infected patients in an Italian primary care setting. Furthermore, the secondary aim is to explore the barriers to HCV treatment.

## 2. Methods 

### 2.1. Study Population and Data Collection

The study was carried out with the active cooperation of 44 GPs (covering an assisted population of 63,955, Campania region), all belonging to the Italian National Health Service (NHS) and affiliated to medical associations (“Samnium medica”, “Progetto Leonardo” and “Cooperativa Moscati”). All the participating GPs use the same software to record data during their daily practice (Millewin; Millenium Dedalus, Florence, Italy) and receive formal periodic training for data entry. For the purpose of this study, data were retrieved using an anonymous encrypted patient code linking demographic details with medical diagnoses, diagnostic tests, prescription of specialist visits and date of death or censoring (transfer to another GP). All diagnoses were coded according to the International Classification of Diseases, 9th Revision, Clinical Modification (ICD-9-CM). 

The study design is based on the GPs review of all subjects, aged 18 or older, with already known diagnosis of HCV infection (ICD9-CM 070.41, 070.44, 070.51, 070.54, 070.7, 070.70, 070.71) or those with a high-risk profile for HCV infection, alive on 30 June 2019 (index date). Patients were defined as “high-risk” if they had at least one of the following criteria at the index date: diagnosis of cirrhosis, chronic liver disease (excluding cirrhosis), acute hepatitis with unknown etiology, HCC, hepatitis B virus (HBV), lymphoma, cryoglobulinemia, hemoglobinopathies, deranged transaminases, patients undergoing hemodialysis or those with positive HCV antibody test but without HCV RNA detection (Table S1 (Supplementary Materials)). 

Between July and October 2019, patients with an already known HCV diagnosis at the index date were reviewed by GPs to determine whether they had received the DAA treatment, and if not, to refer them to specialist care; whereas, patients at high risk for HCV infection were reviewed to determine the positive HCV infection (“patients with newly diagnosed HCV infection”), through HCV antibody and RNA testing, and in that case, to refer them to specialist care for the DAA treatment. 

The results of the analyses were discussed during education and training meetings with hepatology and infectious disease specialists and 250 GPs of the Campania region (including the 44 GPs who were involved in the proactive screening). Knowledge and attitudes to HCV screening and linkage to care were discussed in order to promote either step in the continuum strengthening the communication and collaboration between primary care and physician specialist. In light of this purpose, the GPs reasons for the nonreferral to appropriate specialist care or for the referral recommended by GPs but not performed were explored. 

### 2.2. Statistical Analysis

For both groups, demographic and clinical characteristics were assessed at the index date. Age was categorized into the following groups: <40, 40–49, 50–59, 60–69 and ≥70 years. The presence of signs of chronic liver disease (i.e., clinical signs of fibrosis, cirrhosis or portal hypertension and/or liver enzymes’ derangement) and other clinical conditions such as diabetes (ICD9-CM code 250xx), chronic kidney disease (ICD9-CM code 585xx) and obesity (ICD9-CM code 278xx) were also evaluated at the index date.

Categorical variables were summarized using absolute and relative frequencies. For the univariate analysis we used χ^2^ tests to compare the categorical variables. The proportions of HCV patients already treated, referred and not referred to specialist care were assessed for overall HCV-infected individuals, patients with already known HCV diagnosis and those with a newly diagnosed HCV infection. The analysis was also conducted stratifying all HCV-positive patients in the following two groups: those with signs of advanced liver disease and those without on the basis of clinical features (i.e., clinical signs of fibrosis, cirrhosis or portal hypertension and/or liver enzymes’ derangement).

All analyses were conducted using SPSS software version 23 SPSS Inc., Chicago, IL, USA with *p* < 0.05 indicating statistical significance.

## 3. Results

**Figure 1:**
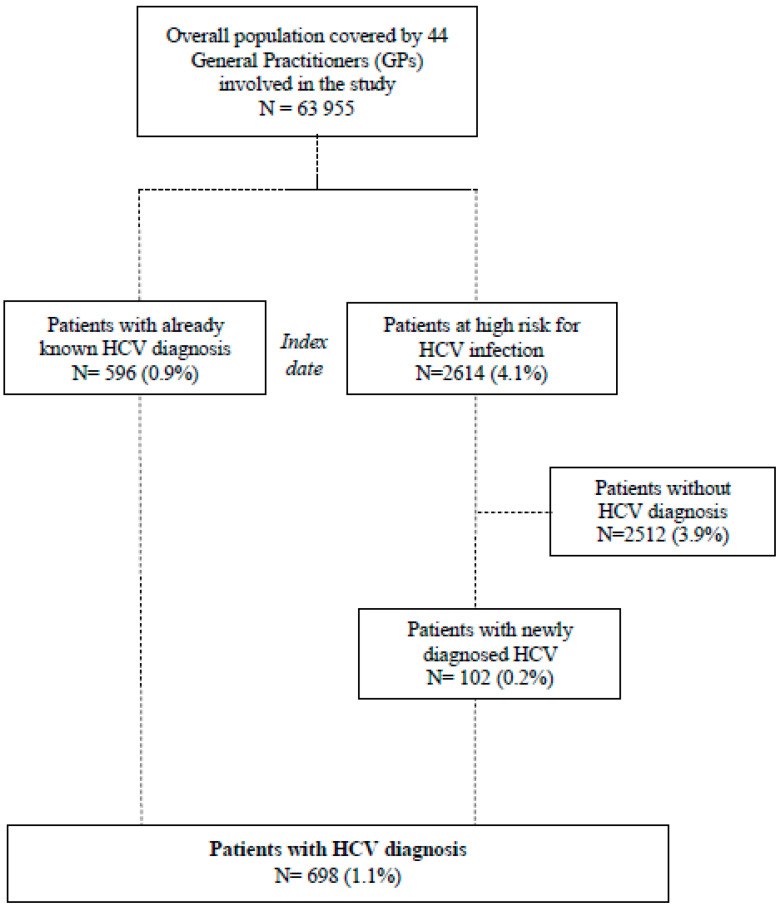
Flow chart of hepatitis C virus (HCV) population.

Overall, 3210 patients were identified, 596 (0.9% of the overall population) patients with already known HCV diagnosis and 2614 (4.1% of the overall population) patients at high risk for HCV infection (Figure 1). With regard to the high-risk group, 79.3% of subjects had a diagnosis of chronic liver disease (excluding cirrhosis), 10.7% deranged transaminases, 5.5% lymphoma and 5.2% HBV (Table S1, Supplementary Materials). Of these, 3.9% (102/2614) were found to have an HCV infection, corresponding to 0.2% (102/63,955) of the total population covered by the GPs. Overall, 698 HCV patients were identified by GPs during the study period (1.1% of the total population), with a mean age of 66.4 years old (Figure 1).

The age distribution was similar for patients with already known HCV diagnosis and those with a newly diagnosed HCV infection (*p* = 0.13) (Table 1). The 49.9% of patients were 70 years old or older, 21.5% between 60 and 69 years and 28.6% below 60 years old. With regards to the other clinical conditions, the differences between the two groups were not statistically significant (Table 1).

**Figure 2:**
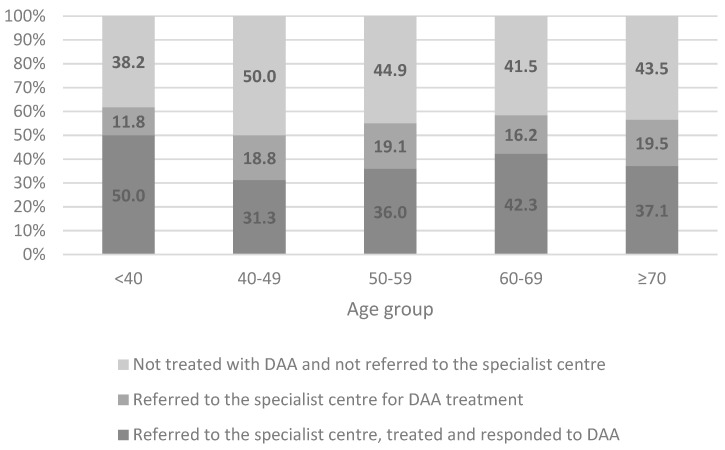
Distribution of patients already treated, referred and not referred to specialist care for direct-acting antiviral (DAA) treatment stratified by age group.

Table 2 reports the frequency of HCV patients already treated, referred and not referred to specialist care for the overall population, patients with known HCV diagnosis and those with a newly diagnosed HCV infection. Of the total of HCV patients, 38.8% were already treated with DAAs, 18.9% were referred to the specialist center and 42.3%, were not sent to specialist care for DAA treatment. Similar proportions were found when we analyzed patients with already known HCV diagnosis and those with a newly diagnosed HCV infection (Table 2). Figure 2 shows the distribution of HCV patients already treated, referred and not referred to specialist care stratified by age groups. The percentage of subjects not treated with DAA and not referred to the specialist center was higher than 40% in patients ≥40 years old. In addition, when we stratified by advanced liver disease, we found that 37.1% of HCV patients without advanced liver disease were already treated with DAAs, 18.4% were referred to the specialist center and 44.5%, were not sent to specialist care; among those with advanced liver disease these frequencies were 42.1%, 19.8% and 38.1%, respectively (Table S2, Supplementary Materials).

Old age, presence of cognitive disorders, ongoing treatment for cancer disease, poor adherence to drug treatment and fear of drug interactions were the causes of nonreferral to specialist care. Patient fear of DAAs side effects following its negative experience with interferons, difficult access to specialist care (e.g., geographic distance from specialist center, long waiting lists) and patient refusal were indicated as the reasons of referral considered by GPs but not performed.

## 4. Discussion

Given the significant potential impact GPs may have towards achieving WHO targets, we conducted this study to support GPs in proactive case-finding of new HCV patients and to assess the linkage to care in the clinical practice. Furthermore, we focus the attention to understand the potential barriers to HCV treatment. Broadly, our findings show that it is possible for GPs, by routinely collecting data, to identify patients at high risk of HCV infection who should be screened and those with already known HCV diagnosis but still not treated. Nevertheless, the study findings highlight that, at least in our area, the screening process should be coupled with educational programs to address HCV-related knowledge gaps among GPs in order to improve the workforce and increase opportunities for HCV screening and engagement into care.

Specifically, we found 698 HCV patients, corresponding to a prevalence of 1.1% in the total population studied. Of these, 102 (15%) were newly diagnosed HCV cases, equal to 0.2% of the total population. The estimate of prevalence of undiagnosed infection highlights that tackling the underdiagnosis of HCV remains a key challenge in eliminating HCV. Similarly, in a study performed in 5 urban areas of Italy, among patients randomly selected from lists of GPs and tested for HCV, 20% of those found HCV RNA positive were unaware of their status [19]. Moreover, we found that it affects mostly the older generations, as previously reported [20]. Indeed, previous studies conducted in Italian rural and peripheral urban areas reported that more than half of patients were over 70 years of age [19,21,22]. This finding may support the corollary that the impact of HCV will significantly decrease in Italy with the next generation, as a consequence of the natural depletion of the predominant elderly infected cohorts [13,19].

Notably, we found that even after identification, approximately 42% of patients were not referred to a specialist center for appropriate investigation and to initiate the proper HCV treatment, and only 39% were treated. Similar results were observed both in patients with an already known HCV diagnosis and those with a newly diagnosed HCV infection. Likewise, a recent study which involved 27 drug dependency centers in the Campania region reports that of 3796 subjects found to be HCV positive, only 20.7% were treated [23]. The disparity between HCV screening and linkage of care were also observed in other countries. A retrospective study of patients screened for HCV within the University of California between February and July 2018 reported that approximately 27% of patients with detectable HCV antibodies were not linked to either further testing or specialty care and specifically young adults and PWID were found as the important predictors of lack of linkage to care [24]. The rate of referral of anti-HCV positive patients for appropriate specialist care was less than 50%, also in a previous UK study [25]. The authors suggest that the reasons for these alarmingly low rates of onward referral and management are multifactorial and complex, reflecting both systems failure and patient choice.

Interestingly, we also found that a considerable proportion of HCV patients without signs of chronic liver disease (44.4%) were not treated and not referred to specialist care for further investigation and management of infection. This evidence underscores missed opportunities for therapeutic intervention before the onset of advanced liver disease just when the expected benefit of treatment should be significantly greater. As reported in previous studies, high hospitalization risk and costs were driven by hepatic and extrahepatic conditions related to HCV disease progression [26]. Indeed, results from a recent Italian experience highlight the potential benefit that early identification and treatment of HCV might have on the reduction of hospitalization costs driven by extrahepatic conditions in addition to liver-related sequelae [27]. HCC is confirmed as the strongest predictor of becoming a high-cost healthcare user, suggesting that as disease severity increases, patients become more costly and resource-consuming [27].

Our analysis is based on the assumption that it is appropriate for all HCV patients identified by GPs to be referred to a specialist for further investigation and management and, therefore, it is crucial to explore the barriers to the linkage to care and HCV treatment. Moreover, our hypothesis is that most obstacles to HCV treatment could be modifiable. Therefore, during the education and training meetings, we investigated the cascade of care following HCV screening and evaluated the reasons for nonreferral and no HCV treatment. Patient’s old age, the presence of comorbid conditions (i.e., cognitive disorders), ongoing treatment for cancer disease, known poor patient adherence for drug interventions and fear of drug interactions were the main causes of GPs nonreferral. On the other hand, patient fear of DAAs side effects following the negative experience with interferons, difficult access to specialist care (e.g., geographic distance from specialist center, long waiting lists) and patient refusal were indicated as reasons for a referral recommended by GPs but not performed. It seems that HCV infection is not always perceived as a major priority, since HCV being perceived as a slowly progressive infection would get less priority for GP or patient than more immediate issues. In light of these data, the knowledge of HCV natural history and HCV treatment should be implemented, indicating the need to educate GPs, at least in our area, about the entire HCV screening to linkage to care process. In accordance with our findings, Samuel at al. illustrated how GPs knowledge of HCV natural history and treatment can influence the screening program [28]. Davis et al. demonstrated that some improvements of GPs in identifying and managing patients with chronic HCV infection can be achieved by means of educational activities involving the learner in a direct and interactive way.

Our results should be interpreted in the context of some limitations. First, the inclusion of GPs not randomly selected but only volunteers may represent a potential source of selection bias. Second, the participating GPs were all from a southern Italian area and therefore, it is not representative of the entire GPs workforce across Italy. Third, GPs may have difficulties in finding previous (if any) test results from electronic records and, additionally, the use of routinely electronic recorded data may have missed patients whose risk factors were not documented (i.e., the presence of blood transfusion in patients’ clinical history) so, the impact of HCV infection on the population covered may be underestimated.

In conclusion, despite the availability of highly effective therapeutic regimens based on direct-acting antivirals, many barriers to HCV eradication still remain. Given that HCV infection is often silent, case-finding needs to be proactive and based on risk information. In this light, GPs workforce may have an important impact on the HCV screening–linkage to care process, hence their engagement is crucial to achieving the goal of HCV eradication. Nevertheless, we suggested that, at least in our area, there needs to be greater outreach, awareness and education among GPs in order to enhance HCV testing, linkage to care and treatment.

## Figures and Tables

**Table 1:** Demographic and clinical characteristics of patients with hepatitis C virus (HCV) diagnosis.

	Overall (N = 698)%	Patients with Already Known HCV Diagnosis (N = 596)%	Patients with Newly Diagnosed HCV (N = 102)%	*p* Value
**Age Groups**				
<40	5.2	5.4	3.5	0.13
40–49	9.7	10.0	8.0	
50–59	13.7	12.4	21.8	
60–69	21.5	22.4	16.1	
≥70	49.9	49.8	50.6	
**Gender**				
Male	48.9	47.4	57.6	0.06
Female	51.1	52.6	42.4	
**Comorbidities**				
Diabetes	14.7	14.3	16.7	0.54
CKD	4.9	4.2	8.8	0.05
Obesity	4.3	3.7	7.8	0.06

Note: HCV, hepatitis C virus; CKD, chronic kidney disease.

**Table 2:** Patients already treated, referred and not referred to specialist care for direct-acting antiviral (DAA) treatment.

	Overall N = 698		Patients with Already Known HCV Diagnosis N = 596	Patients with Newly Diagnosed HCV N = 102	*p* Value
	% ^1^	% ^2^	%	%	
**Referred to the Specialist Center, Treated and Responded to DAA**	0.4	38.8	39.6	33.7	0.26
**Referred to the Specialist Center for DAA Treatment**	0.2	18.9	18.1	23.8	0.18
**Not Treated with DAA and not Referred to the Specialist Center**	0.5	42.3	42.3	42.5	0.96

^1^ Percentage calculated on population covered by 44 general practitioners involved in the study (N = 63,955). ^2^ Percentage calculated on total hepatitis C virus (HCV) patients (N = 698).

## Supplementary Materials

The following are available online at https://www.mdpi.com/2075-1729/10/12/359/s1, Table S1: Criteria for identification of the patients with high risk of hepatitis C virus (HCV), Table S2: High-risk patients stratified by the selection criteria, Table S3. Patients already treated, referred and not referred to specialist care for direct-acting antiviral (DAA) treatment stratified by chronic liver disease.
