# Supplementary material for: Screening, Linkage to Care and Treatment of Hepatitis C Infection in Primary Care Setting in the South of Italy
Source: Life (Basel). 2020 Dec 18;10(12):359. doi: 10.3390/life10120359 (PMC7766029; doi:10.3390/life10120359)
Supplement: Supplementary file 1 [file life-10-00359-s001.pdf]

Table S1. Criteria for identification of the patients with high risk of hepatitis C virus (HCV).

**Criteria of the high-risk hepatitis C virus (HCV) patients**	ICD9-CM codes for diagnosis	Laboratory values	%[1]
Diagnosis of chronic liver disease (chronic hepatitis or alcohol liver disease, nonalcoholic steatohepatitis) excluding cirrhosis	5710, 5713, 5714, 57140, 57141, 57149, 5718, 5719, 5710, 5711		79,3
Elevated transaminases		AST > 68 U/l or/and ALT > 75 U/l	10,7
Diagnosis of lymphoma	201xx e 202xx		5,5
Chronic hepatitis B (HBV) infection	070.2x, 070.3x		5,2
Diagnosis of cirrhosis	571, 5712, 5715, 5716		2,3
Positive hepatitis C antibody test without quantitative HCV RNA test	070.41, 070.44, 070.51, 070.54, 070.7, 070.70, 070.71		1,8
Diagnosis of hepatocellular carcinoma	155		0,5
Human immunodeficiency virus (HIV) infection	042xx, 044xx, 079.53, V08		0,5
Hemoglobinopathies	28261, 28262, 28263, 2827		0,5
Acute and subacute necrosis of liver	570		0,4
Current or prior hemodialysis	V56.0		0,3
Cryoglobulinemia	273.2		0,1

[1] Percentage calculated on patients at high risk of HCV (N=2 614)

Table S2. Patients already treated, referred and not referred to specialist care for direct-acting antiviral (DAA) treatment stratified by chronic liver disease.

	**HCV patients without advanced liver disease**<br>**N = 496**<br>%	**HCV patients with advanced liver disease**<br>**N = 202**<br>%	**p value**
**Referred to the specialist centre, treated and responded to DAA**	37.1	42.1	0.21
**Referred to the specialist centre for DAA treatment**	18.4	19.8	0.67
**Not treated with DAA and not referred to the specialist centre**	44.5	38.1	0.13

Note: HCV, hepatitis C virus.
